# High-Intensity Interval Training in Heart Transplant Recipients: A Systematic Review with Meta-Analysis

**DOI:** 10.5935/abc.20180017

**Published:** 2018-02

**Authors:** Raphael José Perrier-Melo, Fernando Augusto Marinho dos Santos Figueira, Guilherme Veiga Guimarães, Manoel da Cunha Costa

**Affiliations:** 1 Universidade de Pernambuco (UPE), Recife, PE - Brazil; 2 Instituto de Medicina Integral Professor Fernando Figueira, Recife, PE - Brazil; 3 Universidade de São Paulo, São Paulo, SP - Brazil; 4 Universidade de Pernambuco (UPE), Recife, PE - Brazil

**Keywords:** Exercise, Heart Failure/physiopathology, Life Style, Cardiac Rehabilitation, Meta-Analysis as Topic

## Abstract

Heart transplantation (HTx) is considered an efficient and gold-standard procedure for patients with end-stage heart failure. After surgery, patients have lower aerobic power (VO_2_max) and compensatory hemodynamic responses. The aim of the present study was to assess through a systematic review with meta-analysis whether high-intensity interval training (HIIT) can provide benefits for those parameters. This is a systematic review with meta-analysis, which searched the databases and data portals PubMed, Web of Science, Scopus, Science Direct and Wiley until December 2016 (pairs). The following terms and descriptors were used: “*heart recipient*” OR “*heart transplant recipient*” OR ”*heart transplant*” OR “*cardiac transplant*” OR “*heart graft*”. Descriptors via DeCS and *Mesh* were: “*heart transplantation*’’ OR “*cardiac transplantation*”. The words used in combination (AND) were: “*exercise training*” OR “*interval training*” OR “*high intensity interval training*” OR “*high intensity training*” OR “*anaerobic training*” OR “*intermittent training*” OR “*sprint training*”. The initial search identified 1064 studies. Then, only those studies assessing the influence of HIIT on the post-HTx period were added, resulting in three studies analyzed. The significance level adopted was 0.05. Heart transplant recipients showed significant improvement in VO_2_peak, heart rate and peak blood pressure in 8 to 12 weeks of intervention.

## Introduction

Heart transplant (HTx) is considered the gold-standard treatment for patients with heart failure refractory to clinical therapy and/or intervention procedure.^1,2^ The bicaval technique is currently used in surgical centers, consisting in cardiac denervation via complete dissection of the right atrial appendage and interauricular septum, saving a small portion of the left atrial appendage containing the pulmonary veins.^3^ The major advantage of that technique over the others is atrial geometry preservation, lower transpulmonary gradient and lower incidence of post-surgical tricuspid regurgitation.^4^

Cardiac denervation causes cardiorespiratory (maximum oxygen uptake - VO_2_max) and hemodynamic (heart rate - HR, cardiac output - CO and blood pressure - BP) controls to depend initially on the Frank-Starling mechanism (the law states that preload depends on venous return) and, later, on the concentrations of circulating catecholamines and ejection fraction, because of the lack of sympathetic and parasympathetic stimulation and baroreflex.^5-7^ Therefore, transplant recipients have a lower VO_2_max (70-80% of the value predicted for age as compared to healthy individuals),^8^ high levels of HR, BP and vascular resistance at rest. However, physical exercise causes depressed increase in HR and BP, accompanied by an increase in vascular resistance.^9^ This behavior is similar in conditions of submaximal and close-to-peak efforts, causing lower peak HR (HRpeak) and peak BP (BPpeak), with good reproducibility for VO_2_peak. In addition, the post-exercise recovery is slow compared to that of healthy individuals of the same age group.^10,11^

The physiological changes previously mentioned and the immunosuppressive therapy cause cardiorespiratory and hemodynamic damage over time, and transplant recipients often develop diseases, such as systemic arterial hypertension (95%), hyperlipidemia (81%), vasculopathy (50%), kidney failure (33%) and type 2 diabetes mellitus (32%).^12,13^ Thus, cardiac rehabilitation programs have been recommended since the first guidelines of the American Heart Association and American College of Sports Medicine. The major objective of such programs is to re-establish the patients’ daily activities and to change their lifestyle, by adding activities that improve their physical, psychological and social conditions. Those activities should be structurally and continuously performed, focussing on developing the patient’s major deficient variables.^14^ The current guideline recommends that cardiac rehabilitation be composed partially of physical training, consisting of three to five sessions of continuous exercise (walking, jogging, cycling) per week, at mild to moderate intensity, for at least 30 minutes daily.^15,16^ The sessions should begin and end with short warm-up and cool-down periods (5-10 minutes) at low intensity, respectively. Post-HTx physical exercise is safe and effective to promote significant improvement in cardiorespiratory, metabolic, hemodynamic, endothelial and morphological variables.^14,15^

However, studies of systematic review with meta-analysis conducted in patients with coronary artery disease,^16,17^ type 2 diabetes mellitus^18^ and metabolic syndrome^19^ have shown that, in contrast to moderate-intensity continuous training (MICT), high-intensity interval training (HIIT) enables patients to reach similar and/or superior benefits regarding the variables decompensated by those diseases.^20^ The HIIT is characterized by sets of short- or long-lasting exertion periods (30s - 4min) at high intensity (> 85% VO_2_max), followed by short- or long-lasting recovery periods (30s - 4 min).^21^

Although some studies have shown greater progress with HIIT practice as compared to MICT, HIIT is still cautiously prescribed for individuals diagnosed with cardiovascular and metabolic diseases or those who underwent an organ transplantation. In addition, little is known about the dose-response ratio of the improvement in cardiorespiratory, endothelial and hemodynamic parameters caused by HIIT in HTx recipients. Thus, this study was aimed at assessing by use of a systematic review with meta-analysis whether HIIT can benefit those parameters.

## Methods

A systematic review was conducted following the recommendations and meeting the criteria determined by the Preferred Reporting Items for Systematic Reviews and Meta-Analysis guideline (PRISMA).

### Search strategy

The search for articles in English was conduct in the PubMed, Web of Science, Scopus, Science Direct e Wiley databases up to December 2016. The terms and descriptors used in the searching process were selected based on the keywords available in previous studies and via DeCS and Mesh, respectively (Table 1). The terms identified in the literature were: “*heart recipient*” OR “*heart transplant recipient*” OR “*heart* *transplant*” OR “*cardiac transplant*” OR “*heart graft*”. The descriptors of DeCS and Mesh were: “*heart transplantation*’’ OR “*cardiac transplantation*”. The words used in combination (AND) were “*exercise training*” OR “*interval training*” OR “*high intensity interval training*” OR “*high intensity training*” OR “*anaerobic training*” OR “*intermittent training*” OR “*sprint training*”. Data extraction and all processes of search, selection and assessment of articles were performed in pairs.

**Table 1:** Strategy of the bibliographic search in data bases and portals.

#1 "*heart recipient*"[tiab], OR "*heart transplant recipient*"[tiab], OR "*heart transplant*" [tiab], OR "*cardiac transplant*" [tiab], OR "*heart graft*" [tiab], OR"*heart transplantation*''[Mesh], OR "*cardiac transplantation*" [Mesh]	#2 "*exercise training*" [tiab], OR "*interval training*" [tiab], OR "*high intensity interval training*" [tiab], OR "*high intensity training*" [tiab], OR "*anaerobic training*" [tiab], OR "*intermittent training*" [tiab], OR "*sprint training*" [tiab]
#1 AND #2	

Mesh: Medical Subject Headings

### Selection criteria

The inclusion criteria were as follows: a) randomized studies assessing VO_2_peak (based on a maximum incremental test) and/or HRpeak as primary outcome; b) sample comprised exclusively of HTx recipients; c) studies assessing the HIIT effect; and d) studies with an intervention period longer than 4 weeks.

The exclusion criteria were as follows: a) studies without a control group; b) studies with acute analysis; and c) case studies.

### Identification and selection of studies

Initially the references were reviewed based on the titles and abstracts. Then, the relevant articles according to the selection criteria were fully read and assessed regarding their methodological quality by use of the Testex scale.^22^

### Data analysis

The variables analyzed (VO_2_peak and HRpeak) were classified as continuous, and data were presented as mean and standard deviation. Data were combined to obtain the size of the general effect, 95% confidence interval (CI) and significance level, using the Review Manager (RevMan) software, version 5.3, Copenhagen: The Nordic Cochrane Centre. The HIIT group was compared with the control group (post-entrance) by use of weighted mean difference (WMD). For each result, heterogeneity (I^2^) was calculated, adopting the fixed effects model. The significance level adopted was p < 0.05.

## Results

**Figure 1:**
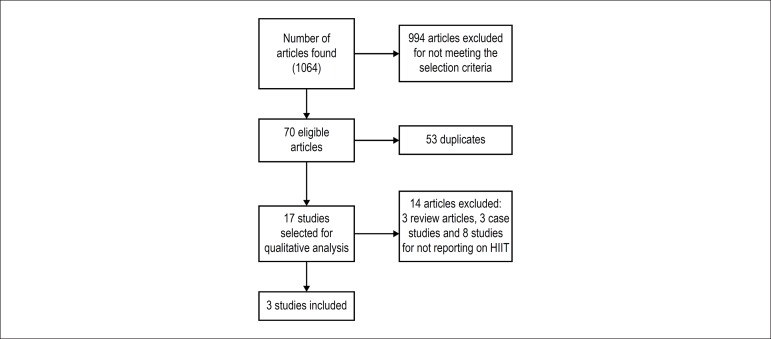
Flowchart of the search and selection process of the articles included in this review.

Figure 1 shows the flowchart of the search and selection process of the articles included in this review.

In the initial electronic search, 1064 potentially relevant studies were identified. After reading their titles, 994 articles were ruled out because they did not have a primary outcome related to the objective of the present review. Then, after reading the abstracts of the remaining studies, 14 were excluded because they did not meet the selection criteria of this study. Three articles with a mean score regarding methodological quality of 10 points, according to the Testex scale, were included in the final analysis.

Major information regarding sample characteristics, methodology, qualitative analysis and results from the studies on HTx recipients are shown in chronological order in Tables 2 and 3. A total of 118 patients (90 men and 28 women) who had undergone HTx 5.3 ± 3.7 years before were included in the analysis of this systematic review, 60 in the HIIT group (49.3 ± 12.7 years) and 58 in the control group (53 ± 14.3 years), maintaining their usual activities. The HIIT sessions were conducted on cycle ergometers^23,24^ and treadmills,^25^ reaching an intensity of 80-100% of VO_2_peak or 85-95% of HRmax. Such training sessions were performed three to five times per week for 8 and 12 weeks.

**Table 2:** Characteristics of the sample, methodological quality and major results of the studies assessing the effect of high-intensity interval training (HIIT) on heart transplant (HTx) recipients.

Study	GROUPS		HIIT protocol	Duration (weeks)	Major results	Testex														
	**HIIT**	**CONTROL**				**1**	**2**	**3**	**4**	**5**	**6**	**6**	**6**	**7**	**8**	**8**	**9**	**10**	**11**	**12**
Haykowsky et al., 2009	N = 2217M/5F57 ± 10 Post-HTx time = 5.4 ± 4.9 years	N = 2118M/3F59 ± 11 Post-HTx time = 4.4 ± 3.3 years	Cycle ergometer and treadmill**1-8 weeks** 5x/week 30-45 min: 60-80%VO_2_peak	5x/week 12 weeks	12 weeks of training significantly increased VO_2_peak (21.2 ± 7.3 - 24.7 ± 8.8 mL/kg/min, p = 0.003) of HTx recipients	**+**	**+**	**+**			**+**	**+**			**+**	**+**	**+**		**+**	**+**
			**9-12 weeks**3x/weeks 30-45 min: 60-80%VO_2_ peak2x/weeks 20-25x (30s: 90-100% VO_2_peak/1 min)																	
Hermann et al., 2011	N = 1412M/2F53 ± 11Post-HTx time = 6.8 ± 4.0 years	N = 1310M/3F47 ± 18Post-HTx time = 7.0 ± 5.5 years	Cycle ergometer and staircase running4 min: 80% VO_2_ peak/ ½ min2 min: 85% VO_2_peak/ ½ min30 s: 90% VO_2_peak/ ½ min	3x/week 8 weeks	The 8-week HIIT program significantly reduced SBP (p = 0.02) and significantly increased VO_2_peak (p < 0.001) and endothelial action	**+**	**+**	**+**			**+**	**+**			**+**	**+**	**+**		**+**	**+**
Nytroen et al., 2012	N = 2416M/8F48 ± 17Post-HTx time = 4.3 ± 2.4 years	N = 2417M/7F53 ± 14Post-HTx time = 3.8 ± 2.1 years	Treadmill4 min (85-95% HRmax) / 3 min (11-13 Borg SEP)	3x/week 8 weeks	HIIT significantly improved VO_2_peak (p < 0.001) after 8 weeks of training	**+**	**+**	**+**			**+**	**+**			**+**	**+**	**+**		**+**	**+**

N: sample; M: male; F: female; HRmax: maximum heart rate; SEP: subjective effort perception; SBP: systolic blood pressure.

**Table 3:** Major results of the hemodynamic and cardiorespiratory variables found in the studies

	HIIT		CON		
**VARIABLES**	**Pre**	**Post**	**Pre**	**Post**	**Studies**
HR at rest	-	-	-	-	Haykowsky et al., 2009
	76 ± 11	76 ± 7 (NS)	78 ± 7	78 ± 11 (NS)	Hermann et al., 2011
	85 ± 11	83 ± 11 (NS)	79 ± 11	81 ± 13 (NS)	Nytroen et al., 2012
HRpeak	147 ± 18	154 ± 15 (0.06)	139.6 ± 19	139 ± 20 (NS)	Haykowsky et al., 2009
	-	-	-	-	Hermann et al., 2011
	159 ± 14	163 ± 13 (< 0.05)	154 ± 15	153 ± 17 (NS)	Nytroen et al., 2012
VO_2_peak	21.2 ± 7.3	24.7 ± 8.8 (0.03)	18.2 ± 5.9	18.2 ± 5.3 (NS)	Haykowsky et al., 2009
	23.9 ± 6.7	28.3 ± 6.1 (< 0.001)	24.6 ± 5	23.4 ± 5.7 (NS)	Hermann et al., 2011
	27.7 ± 5.5	30.9 ± 5.3 (< 0.001)	28.5 ± 7	28 ± 6.7 (NS)	Nytroen et al., 2012
FMD	4 ± 6.8	5.3 ± 4.9 (NS)	3.2 ± 4	3.9 ± 5.2 (NS)	Haykowsky et al., 2009
	8.3 ± 1.3	11.4 ± 1.2 (0.01)	5.6 ± 1	5.3 ± 1.7 (NS)	Hermann et al., 2011
	-	-	-	-	Nytroen et al., 2012
SBP	-	-	-	-	Haykowsky et al., 2009
	142 ± 17	127 ± 13 (0.02)	141 ± 15	142 ± 23 (NS)	Hermann et al., 2011
	130 ± 17	136 ± 16 (NS)	131 ± 20	129 ± 14 (NS)	Nytroen et al., 2012
DBP	-	-	-	-	Haykowsky et al., 2009
	85 ± 7	82 ± 9 (NS)	82 ± 9	84 ± 14 (NS)	Hermann et al., 2011
	80 ± 10	82 ± 9 (NS)	81 ± 15	82 ± 17 (NS)	Nytroen et al., 2012
SBPpeak	175 ± 26	177 ± 21 (NS)	172 ± 29	180 ± 27 (NS)	Haykowsky et al., 2009
	-	-	-	-	Hermann et al., 2011
	181 ± 33	211 ± 66 (< 0.05)	197 ± 22	191 ± 32 (NS)	Nytroen et al., 2012
DBPpeak	81 ± 9	79 ± 9 (NS)	81 ± 8	80 ± 9 (NS)	Haykowsky et al., 2009
	-	-	-	-	Hermann et al., 2011
	71 ± 15	80 ± 14 (< 0.05)	83 ± 14	91 ± 35 (NS)	Nytroen et al., 2012

HIIT: high-intensity interval training; HR: heart rate; FMD: flow mediated dilation of the brachial artery; SBP: systolic blood pressure; DBP: diastolic blood pressure; NS: nonsignificant.

**Figure 2:**
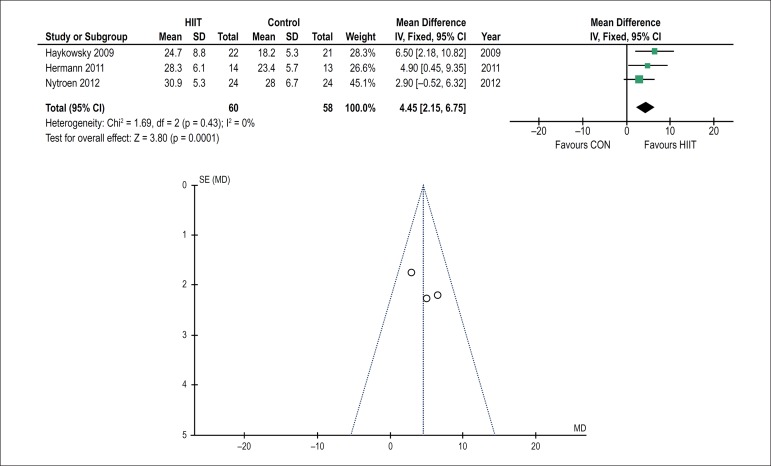
Forest plot (A) AND funnel plot (B) showing information about the effect of high-intensity interval training (HIIT) on VO_2_peak.

**Figure 3:**
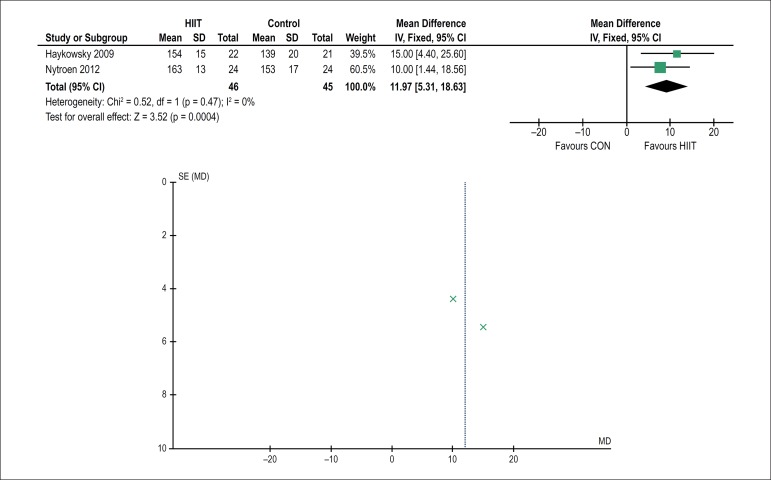
Forest plot (A) AND funnel plot (B) showing information about the effect of high-intensity interval training (HIIT) on peak heart rate.

All studies included had VO_2_peak as the major outcome of the analysis. Figure 2 shows the increased effect on VO_2_peak [95%CI: 4.45 (2.15 - 6.75), p = 0.0001, N = 118] of HIIT (24.3 ± 6.5 - 28.0 ± 6.7 mL/kg.min; 15%) as compared to that of the control group (23.8 ± 6.0 - 23.2 ± 5.9 mL/kg.min; -2%). Regarding HRpeak, based on the comparative analysis of the groups, two studies reported a favorable effect [95%CI: 0.74 (0.31 - 1.16) p = 0.0007, N = 46] in the HIIT group (Figure 3).

The studies that were not statistically analyzed (forest plot) showed, in the HIIT group, a positive effect on BP at rest and BPpeak (systolic and diastolic), brachial flow velocity, maximal muscle strength (1 RM), lean mass maintenance, and inflammatory markers. Some of those results are shown in Table 3. In addition, none of the studies reported a cardiovascular event and/or mortality associated with training, showing it to be a safe practice to be included in cardiac rehabilitation programs.

## Discussion

The present systematic review with meta-analysis is the first to analyze the effect of HIIT on some health-related parameters of HTx recipients. The three studies included showed that HIIT improved VO_2_peak by 15%. Such increase is greater than that found in two systematic reviews with meta-analysis that assessed the effect of different types of exercise^26^ and of MICT^27^ on the VO_2_peak of those patients.

Although HIIT improves VO_2_peak, sometimes it is not indicated for HTx recipients because they have chronotropic insufficiency developed from cardiac denervation.^28^ That incompetence hinders HR at rest (increase) and during close-to-peak exercise (decrease - HRpeak), decreasing the chronotropic reserve values. Thus, according to the studies assessed in this review, 8 to 12 weeks of HIIT intervention can decrease HR at rest and increase HRpeak. High-intensity exercise (> 80%VO_2_peak or > 85%HRmax) might have improved the cardiocirculatory function, stimulating the sinus node faster, facilitating faster and better responses on HR at rest and HRpeak.^29^

Although the literature shows an insufficient number of studies on HIIT and HTx recipients, that type of training can provide significant central and peripheral benefits to improve the clinical findings after surgery.^30^ In addition, recent studies comparing the contribution of HIIT and MICT to the deficient variables of HTx recipients have shown the superior effect of HIIT.^31,32^ Such results can indicate a possible change in paradigm regarding the recommendation of exercise prescription for HTx recipients. Thus, further studies are required to identify which training protocol better improves the deficient variables of those patients.

## Conclusion

Our results showed that 8 to 12 weeks of cardiac rehabilitation with HIIT were sufficient to significantly increase HRpeak and aerobic power of HTx recipients (men and women).
